# Metabolomic disorders caused by an imbalance in the gut microbiota are associated with central precocious puberty

**DOI:** 10.3389/fendo.2024.1481364

**Published:** 2024-12-02

**Authors:** Chunjie Liu, Shasha Zhou, Yan Li, Xiaoqin Yin, Pin Li

**Affiliations:** Department of Endocrinology, Shanghai Children’s Hospital, School of Medicine, Shanghai Jiao Tong University, Shanghai, China

**Keywords:** central precocious puberty, gut microbiota, 16S rDNA, metabolomics, GnRH

## Abstract

**Background:**

Central precocious puberty (CPP) is characterized by the premature activation of the hypothalamic-pituitary-gonadal axis, resulting in early onset of sexual development. The incidence of CPP has been rising in recent years, with approximately 90% of cases lacking a clearly identifiable etiology. While an association between precocious puberty and gut microbiota has been observed, the precise causal pathways and underlying mechanisms remain poorly understood. The study aims to investigate the potential mechanisms through which gut microbiota imbalances may contribute to CPP.

**Methods:**

In this study, clinical information and fecal samples were collected from 50 CPP patients and 50 healthy control subjects. The fecal samples were analyzed by 16S rDNA sequencing and UPLC−MS/MS metabolic analysis. Spearman correlation analysis was used to identify the relationships between gut microbiota and metabolites.

**Results:**

The gut microbiota composition in CPP patients was significantly different from that in healthy controls, characterized by an increased abundance of *Faecalibacterium* and a decreased abundance of *Anaerotruncus*. Additionally, significant differences were observed in metabolite composition between the CPP and control groups. A total of 51 differentially expressed metabolites were identified, with 32 showing significant upregulation and 19 showing significant downregulation in the CPP group. Furthermore, Spearman correlation analysis indicated that gut microbiota dysbiosis may contribute to altered metabolic patterns in CPP, given its involvement in the regulation of several metabolic pathways, including phenylalanine and tyrosine biosynthesis and metabolism, the citrate cycle (TCA cycle), glyoxylate and dicarboxylate metabolism, and tryptophan metabolism.

**Conclusions:**

The study revealed the gut microbial and metabolite characteristics of CPP patients by integrating microbiome and metabolomics analyses. Moreover, several key metabolic pathways involved in the onset and progression of CPP were identified, which were regulated by gut microbiota. These findings broaden the current understanding of the complex interactions between gut microbial metabolites and CPP, and provide new insights into the pathogenesis and clinical management of CPP.

## 1 Introduction

Precocious puberty is defined as the onset of secondary sexual characteristics in girls before the age of 8 and in boys before the age of 9. According to its pathogenesis, precocious puberty can be classified into the following three types: central precocious puberty (CPP), peripheral precocious puberty (PPP) and partial precocious puberty. Most cases of CPP are categorized as idiopathic CPP (ICPP), as they lack identifiable predisposing factors. CPP results from the premature activation of hypothalamic-pituitary-gonadal (HPG) axis due to increased secretion of gonadotropin-releasing hormone (GnRH) from the hypothalamus, leading to earlier sexual development. In recent years, the prevalence of CPP has risen significantly, up to 0.5-2% in China, with a higher incidence in girls than in boys (1–3). CPP seriously affects the growth and mental health of children and has attracted attention in both society and the medical community. Therefore, conducting research on the etiology and pathogenesis of ICPP will help us to better understand the disease and establish a foundation for its early diagnosis and treatment.

The pathogenesis of ICPP is complex and multifaceted, which may be the result of a combination of genetic, metabolic and environmental factors. Previous studies have shown that genetic factors play an significant role in the onset and progression of ICPP. Kisspeptin, encoded by the *KISS1* gene, interacts with hypothalamic GnRH neurons via binding to the G protein-coupled receptor GPR54. This interaction stimulates GnRH-dependent secretion of luteinizing hormone (LH) and follicle-stimulating hormone (FSH), initiating the onset of puberty (4, 5). Other known genes associated with ICPP, such as thyroid-specific transcription factor-1 (*TTF1*) and cut homeobox-1 (*CUX1*), were identified in our previous studies (6, 7). *TTF1* encodes a transcription termination factor. *CUX1* encodes a member of the homeodomain family of DNA-binding proteins. These genes are thought to regulate sexual development by modulating the Kiss1/GPR54 system. However, their effects appear to be transient and insufficient to fully control the activation of GnRH neurons (6, 7). In addition, the potential role of environmental factors, such as environmental endocrine disruptors, in increasing the incidence of CPP by affecting the HPG axis remain an important area of investigation (8). Recent findings indicate that obese girls are at a higher risk of developing CPP, suggesting that energy or amino acid metabolic pathways may regulate the hypothalamic neuroendocrine network, thereby prompting the activation of GnRH neurons and contributing to the onset of CPP. However, the precise mechanisms underlying these associations remain unclear, highlighting the need for further research and exploration.

The gut microbiota refers to the collection of microorganisms residing in the human intestine. Previous studies have shown associations between the gut microbiota and various conditions, including diabetes, obesity, Alzheimer’s disease, depression. Additionally, the gut microbiota is closely connected to the neuroendocrine system and plays a crucial role in the brain-gut-microbiome axis (9–12). Exploring the relationship between metabolic disorders resulting from gut microbiota imbalances and host diseases is crucial for advancing disease prevention and treatment. In recent years, the relationship between the gut microbiota, its metabolites and sexual development has attracted attention from researchers (13, 14). In our previous research, we identified that there are three major metabolic pathways - catecholamine metabolism, serotonin metabolism and the tricarboxylic acid cycle - that were altered in children with CPP based on their urine sample analyses. Significant changes were observed in the urinary levels of 4-hydroxyphenylacetic acid, 5-hydroxyindoleacetic acid, indoleacetic acid, 5-hydroxytryptophan, and 5-hydroxykynurenamine in the CPP group. These findings suggested that the development of CPP may be related to metabolic disorders resulting from alterations in the gut microbiota (15). However, the precise causal relationships and underlying mechanisms linking these metabolic disturbances to CPP remain elusive.

In this study, 16S rDNA high-throughput sequencing revealed that the main differences in gut microbiota composition between patients with CPP and healthy controls were an increased abundance of *Faecalibacterium* and a decreased abundance of *Anaerotruncus* at the genus level. Metabolomic analysis further demonstrated significant differences in metabolite composition between the CPP and control groups. A total of 51 differentially expressed metabolites were identified, with 32 showing significant upregulation and 19 showing significant downregulation in the CPP group. Further application of Spearman correlation analysis showed that imbalances in gut microbiota can affect the metabolic patterns in CPP patients, as the gut microbiota is involved in regulating phenylalanine and tyrosine biosynthesis and metabolism, the citrate cycle (TCA cycle), glyoxylate and dicarboxylate metabolism, and tryptophan metabolism. Our findings provide novel insights into the mechanism underlying the onset and progression of CPP.

## 2 Materials and methods

### 2.1 Patients and samples

In our study, a total of 50 stool and serum samples were collected from girls diagnosed with ICPP at Shanghai Children’s Hospital, affiliated to Shanghai Jiao Tong University. Meanwhile, stool and serum samples were collected from 50 healthy children matched with the ICPP group by age, gender, ethnicity and region during the same period. The study was approved by the ethics committee of Shanghai Children’s Hospital, and informed consent was obtained from all participants.

Fresh stool samples were immediately frozen at -80°C to prevent degradation from repeated freeze-thaw cycles. Peripheral blood samples (4 mL) were obtained from each participant, centrifuged at 3000 rpm for 10 minutes, after which the serum was collected, aliquoted into 0.5 mL portions, and stored at -80°C.

### 2.2 DNA extraction, polymerase chain reaction amplification, and Illumina MiSeq sequencing

Microbial DNA was extracted from CPP and control stool samples using the Fast DNA Stool Mini Kit (51604, Qiagen, Germany), according to its instruction manual. Universal primers 341F and 806R were used to amplify the V3-V4 region of the bacterial ribosomal 16S rDNA gene. When designing specific primers, the index sequence and connector sequence suitable for Illumina MiSeq PE250 should be added to the 5’ end of the universal primer. The primer sequences used are as follows:

Forward primer (5’-3’): CCTACGGGRSGCAGCAG (341F)

Reverse primer (5’-3’): GGACTACVVGGGTATCTAATC (806R)

PCR amplification was performed using Kapa Hifi Hotstart Readymix PCR kit with high fidelity enzyme. Amplicons were extracted from 2% agarose gels and purified with AxyPrep DNA gel recovery kit (Axygen Biosciences, USA). The purified PCR products were tested by Thermo Nanodrop 2000 microspectrophotometer and 2% agarose gels.

### 2.3 16S rDNA gene sequence analysis

Qubit 2.0 (Invitrogen, USA) was used for library quantitation. Paired-end sequencing was performed using Illumina’s MiSeq PE250 Sequencer (Illumina, USA). Paired-end data obtained by sequencing was spliced with PANDAseq software (https://github.com/neufeld/pandaseq, version 2.9), and long Reads with high variability were obtained for 16S analysis. The resulting raw reads were filtered as follows: 1) maximum number of N base = 3; 2) minimum average quality score of each read = 20; 3) the length of reads between 250bp and 500bp. Clean Reads are finally obtained. The reads with 97% identity were clustered into Operational Taxonomic Units (OTUs) using UPARSE (http://drive5.com/uparse/). A representative sequence of each OTU was assigned to a taxonomic level in the Ribosomal Database Project (RDP, http://rdp.cme.msu.edu/) database using 0.8 as the minimum confidence threshold. Alpha and beta diversity were calculated using QIIME software (version 1.9.1) with the default parameters. α-diversity represents an analysis of diversity in a single sample reflected by parameters including Observed species index, Chao 1 index, Simpson index, Shannon index and PD whole tree index using QIIME. β-diversity is used to measure the microbiota structure between different groups. The results of Unifrac are used to measure β-diversity, which are generally divided into Unweighted Unifrac and Weighted Unifrac. Both the weighted and unweighted Unifrac distance matrices were plotted in the principal coordinate analysis (PCoA), and analyses of similarities (ANOSIMs) were performed. The higher the index, the greater the differences between groups. The linear discriminant analysis (LDA) effect size (LEfSe) method was used to analyze the differentially expressed bacterial taxa at different levels between CPP patients and healthy controls. LEfSe analysis is mainly used to find and identify two or more biomarkers and genomic characteristics, such as genes, metabolic pathways and taxonomy. LEfSe analysis used LDA to detect differential abundance and characteristics between groups at the phylum, class, order, family, and genus levels. Bacterial taxa with LDA scores greater than the set threshold (the lowest was 2) were considered biomarkers with statistical differences. The abundances of functional categories in the Kyoto Encyclopedia of Genes and Genomes (KEGG) orthologs was predicted by Phylogenetic Investigation of Communities by Reconstruction of Unobserved States (PICRUSt).

### 2.4 Quantitative analysis of microbial metabolomics

Feces samples were thawed on ice-bath to diminish degradation. About 10 mg of each sample was weighted and transferred to a new 1.5 ml tube. Then 25 μl of water was added and the sample was homogenated with zirconium oxide beads for 3 min. 185 μl of ACN/Methanol (8/2) was added to extract the metabolites. The sample was centrifuged at 18000 g for 20 min. Then the supernatant was transferred to a 96-well plate. The following procedures were performed on a Biomek 4000 workstation (Biomek 4000, Beckman Coulter, USA). 20 μl of freshly prepared derivative reagents was added to each well. The plate was sealed and the derivatization was carried out at 30°C for 60 min. After derivatization, 350 μl of ice-cold 50% methanol solution was added to dilute the sample. Then the plate was stored at -20°C for 20 minutes and followed by 4000 g centrifugation at 4°C for 30 min. 135 μl of supernatant was transferred to a new 96-well plate with 15 μl internal standards in each well. Serial dilutions of derivatized stock standards were added to the left wells. Finally, the plate was sealed for LC-MS analysis.

An ultra-performance liquid chromatography coupled to tandem mass spectrometry (UPLC-MS/MS) system (ACQUITY UPLC-Xevo TQ-S, Waters Corp., Milford, MA, USA) was used to quantitate the microbial metabolite in this study by Metabo-Profile Biotechnology (Shanghai) Co., Ltd. The optimized instrument settings are briefly described as follows. For HPLC, column: ACQUITY HPLC BEH C18 1.7 × 10−6 m VanGuard precolumn (2.1 × 5 mm) and ACQUITY HPLC BEH C18 1.7 × 10−6 m analytical column (2.1 × 100 mm), column temp.: 40°C, sample manager temp.: 10°C, mobile phases: A = water with 0.1% formic acid; and B = acetonitrile/IPA (70:30), gradient conditions: 0–1 min (5% B), 1–11 min (5–78% B), 11–13.5 min (78–95% B), 13.5–14 min (95–100% B), 14–16 min (100% B), 16–16.1 min (100-5% B), 16.1–18 min (5% B), flow rate: 0.40 mL min−1, and injection vol.: 5.0 μL. For mass spectrometer, capillary: 1.5 (ESI+), 2.0 (ESI-) Kv, source temp.: 150°C, desolvation temp.: 550°C, and desolvation gas flow: 1000 L h−1.

The metabolites were identified using the *STD* method, employing the Q300 kit (Metabo-Profile, Shanghai, China). This method enables the quantitative detection of a wide array of metabolites, including amino acids, phenols, phenyl or benzyl derivatives, indoles, organic acids, fatty acids, sugars, and bile acids in biological samples of varying concentrations on the same microtiter plate. The Q300 kit utilizes 60 internal standards, such as L_Arginine_15N2, Hippuric acid_D5, TCDCA_D9, D_Glucose_D7, Carnitine_D3, C5 0_D9 and Citric acid_D4, along with 306 one-to-one standards for accurate quantification. The derivatization reaction was carried out using 3-nitrophenylhydrazine as the derivatization reagent and 1-(3-dimethylaminopropyl)-3-ethylcarbodiimide as the catalyst.

Quality control (QC) on the samples were carried out in order to ensure high quality analysis of samples by the instrument. The raw data files generated by UPLC-MS/MS were processed using the QuanMET software (v2.0, Metabo-Profile, Shanghai, China) to perform peak integration, calibration, and quantitation for each metabolite. Mass spectrometry-based quantitative metabolomics refers to the determination of the concentration of a substance in an unknown sample by comparing the unknown to a set of standard samples of known concentration (i.e., calibration curve).

For many metabolomics studies, two types of statistical analysis are extensively performed: 1) multivariate statistical analyses such as principal component analysis (PCA), partial least square discriminant analysis (PLS-DA), orthogonal partial least square discriminant analysis (OPLS-DA) and so on; 2) univariate statistical analyses including student t-test, Mann-Whitney-Wilcoxon (U-test), ANOVA, correlation analysis, etc. PCA is an unsupervised modeling method commonly used to detect data outliers, clustering, and classification trends without *a priori* knowledge of the sample set. The first principal component (PC1) expresses more variation than the second principal component (PC2), which, in turn, expresses more variation than PC3, and so on. PLS-DA and/or OPLS-DA has been extensively used for multi-class classification and identification of differently altered metabolites. In the current project, PLS-DA modeling is used as a multi-class classifier to visualize the difference between global metabolic profiles among the groups that provides more valuable information beyond what can be gleaned from PCA. The OPLS method is an improved PLS-DA method for modeling and further screening of differential metabolites between the CPP group and the control group.

### 2.5 Serum sex hormone detection

Serum samples (25 μl) were analyzed to measure levels of LH, FSH, E2 and other hormones using a chemiluminescence method. The preparation, calibration, dilution, quality control, correction, and analysis procedures were conducted in strict accordance with the operation manual of chemiluminescence instrument (Beckman, USA).

### 2.6 Spearman correlation analysis

Spearman correlation analysis was performed on the 16S rDNA sequencing and metabolomics data to investigate associations between differential gut microbiota and metabolites. LDA >2 and *P* < 0.05 were used as criteria for screening and extracting differential gut microbiota and related functional data, followed by extraction of differential metabolomics data. The results of the Spearman correlation analysis were visualized in a heatmap. For each metabolite, data were included in the heatmap if its correlation with at least one gut microbiota had a *P* value < 0.05 and an absolute correlation coefficient (R) > 0.3.

### 2.7 Statistical analysis

SAS software (Version 9.2) was used for statistical analysis in this study. Age and body mass index (BMI) data between the two groups were compared using the Mann-Whitney Wilcoxon test, with statistically significance defined as *P* < 0.05.

## 3 Results

### 3.1 Clinical data

A total of 50 children with ICPP were recruited from Shanghai Children’s Hospital. The inclusion criteria were as follows: (1) onset of secondary sexual characteristics in girls before 8 years of age; (2) GnRH stimulation test showing a peak LH level (LHP) ≥ 5 mIU/mL and an LHP/FSHP ratio > 0.6; (3) ovarian volume ≥ 1 mL; (4) exclusion of secondary CPP due to other causes; and (5) no history of drug treatment related to CPP, including Chinese herbal medicines. The exclusion criteria were: (1) presence of pituitary tumors or other organic lesions; (2) use of traditional Chinese medicine within 1 month prior to enrollment; (3) use of antibiotics, probiotics or prebiotics within 1 month prior to enrollment; and (4) coexisting gastrointestinal diseases or impaired liver function. At the same time, 50 healthy children matched with the CPP group in age, sex, ethnicity, and region were recruited as controls. None of the participants in either group had a history of other diseases. The mean age of the children in the CPP group was 8.137 years, while the mean age of the control group was 7.902 years. The average BMI of the CPP group was 16.294 kg/m^2^, compared to 15.720 kg/m^2^ for the control group. There were no significant differences in both age and BMI between the two groups (P > 0.05).

### 3.2 Discrepancies in the structure and diversity of the gut microbiota between CPP and control groups

**Figure 1:**
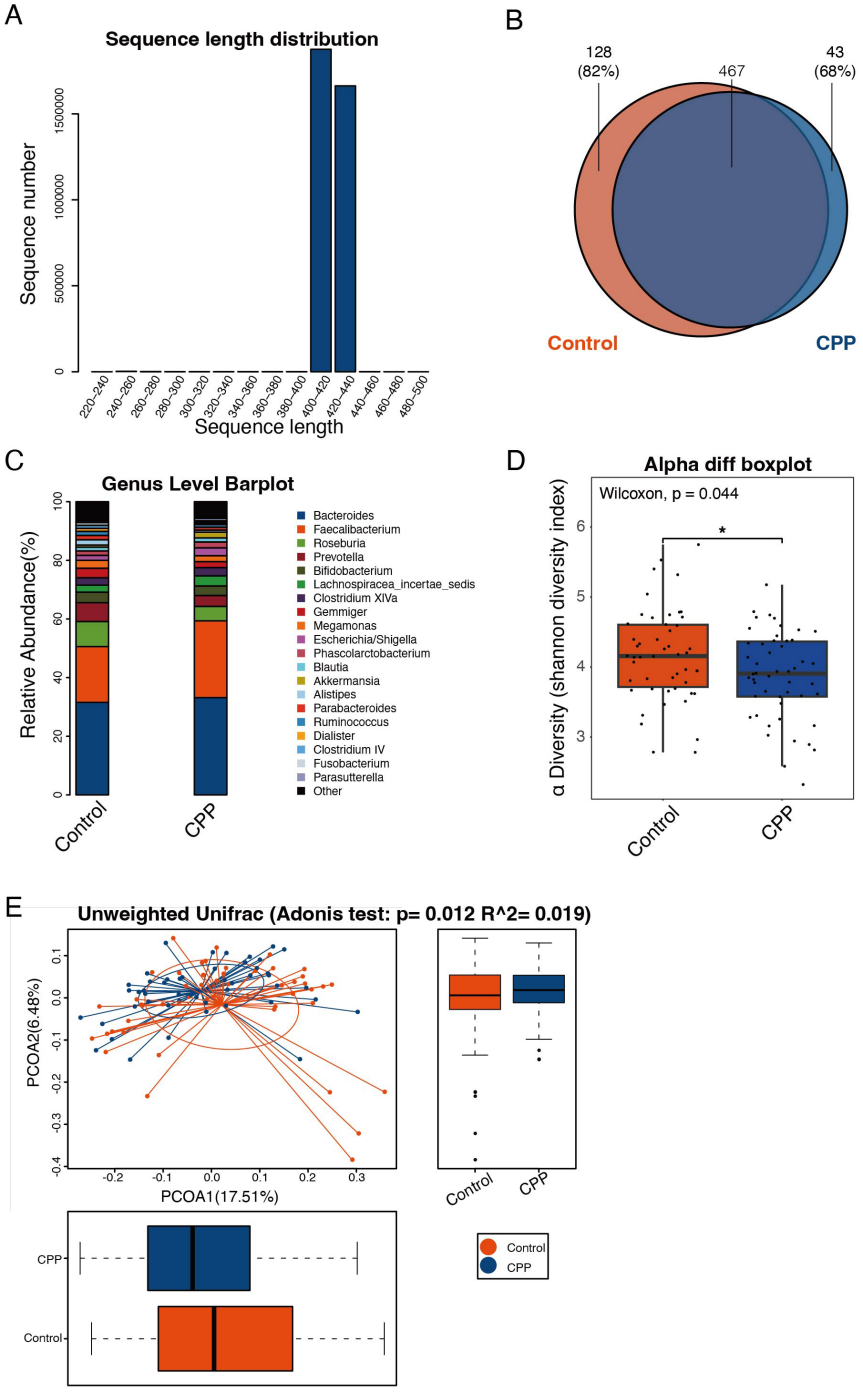
Discrepancy in the structure and diversity of the gut microbiota in the CPP and control groups. **(A)** The sequence distribution across length intervals. Read length was kept within the 250-500 bp range, with those shorter than 250 bp excluded. The X-axis represents sequence length, and the Y-axis shows the count of reads. **(B)** Venn diagram of OTUs. Each color corresponds to a specific group. The overlapping region represents OTUs shared by both groups, while the non-overlapping areas represent unique OTUs in each group. **(C)** Histogram of microbial abundances in the CPP group and control group at the genus level. **(D)** Comparison of the α-diversity index (Shannon index) between the CPP and control group. The X-axis represents the groups, and the Y-axis represents the Shannon index. The box plot presents 5 statistics: minimum, first quartile, median, third quartile, and maximum. An asterisk “*” means 0.01<*p*<0.05. **(E)** Adonis analysis (unweighted UniFrac analysis). The X-axis represents the first principal coordinate, and the Y-axis represents the second principal coordinate. Percentage refers to the contribution of the corresponding principal coordinate to the difference of samples; the points represent individual samples; the horizontal and vertical box plots represent value distributions of the two groups on corresponding principal coordinates.

To explore the correlation between CPP and gut microbiota, fecal samples from girls diagnosed with CPP and healthy controls were analyzed using 16S rDNA high-throughput sequencing. A total of 3,545,161 effective sequences were obtained, with an average of 35,451.61 ± 1,883.29 tags per sample, ranging from 30,067 to 38,945 tags. Sequence lengths were predominantly between 407 to 422 bp, with an average length of 412.65 ± 3.01 bp (**Figure 1A**). Sequences clustered at 97% similarity yielded 638 OTUs, with 467 OTUs shared by both the CPP and control groups. In addition, the results showed that 128 OTUs were unique to the control group, corresponding to 41 individuals (82% of the control subjects), while 43 OTUs were unique to the CPP group, corresponding to 34 patients (68% of CPP patients), as shown in the Venn diagram (**Figure 1B**). These results suggested significant differences in OTU distribution between the two groups.

A representative sequence of each OTU was assigned to a taxonomic level in the RDP. The microbial abundances of the two groups at the phylum, class, order, family and genus levels were analyzed. The results showed that at the genus level, the abundance of *Faecalibacterium* in the CPP group was higher than that in the control group, whereas the abundances of *Prevotella* and *Roseburia* were reduced in the CPP group (**Figure 1C**).

To assess the differences in the diversity and richness of the gut microbiota in the CPP and control group, we analyzed the α-diversity index. The Shannon index showed that the diversity and richness of the gut microbiota in the CPP group were significantly lower than in the control group (*P* = 0.044) (**Figure 1D**). α-diversity analysis, combined with PCoA, indicated substantial differences in fecal microbial composition between CPP patients and controls (Adonis *P* = 0.012, R^2^ = 0.019) (**Figure 1E**).

**Figure 2:**
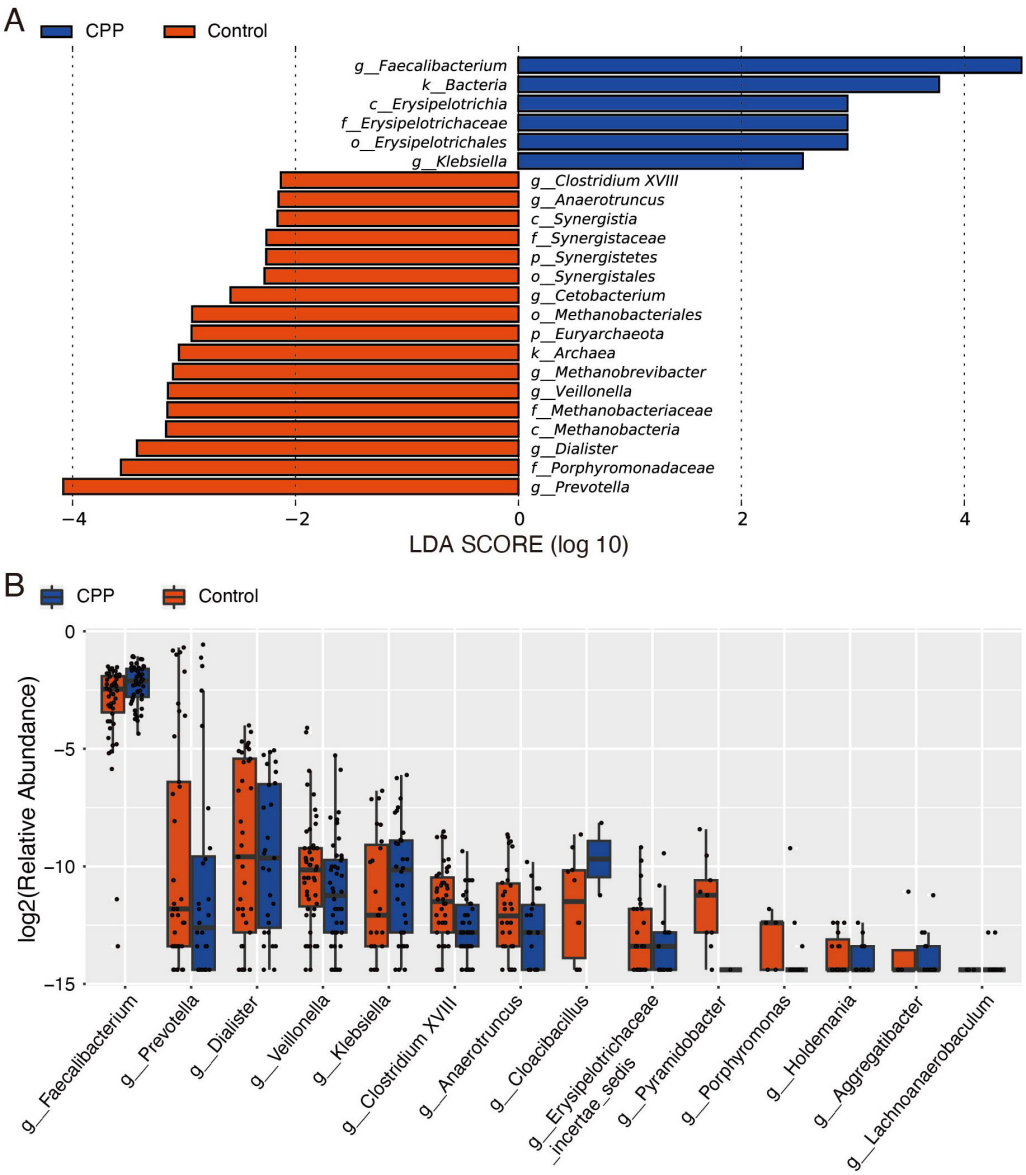
Analysis of different microbiota between the CPP and control groups. **(A)** LEfSe analysis. The LDA score was used to detect differential abundance between the two groups at the phylum, class, order, family, and genus levels. Bacterial taxa with LDA scores above the threshold (minimum of 2) were considered biomarkers with significant differences. Red represents the control group, and blue represents the CPP group. **(B)** The Wilcoxon test was used to analyze different microbiota constituents at the genus level. The X-axis shows genera name, and the Y-axis shows the log2 value of relative abundance.

Further, we carried out LEfSe and Wilcoxon tests to identify specific microorganisms in the gut microbiota that differed between CPP and control groups. Features with an LDA score cut-off of 2 were considered significant. LEfSe analysis showed that, at the phylum level, *Synergistetes* and *Euryarchaeota* were less abundant in the CPP group compared to the control group. At the genus level, *Faecalibacterium* and *Klebsiella* were significantly abundant in the CPP group, while *Prevotella, Anaerotruncus, Dialister, Veillonella, Methanobrevibacter, Cetobacterium* and *Clostridium XVIII* were significantly reduced in the CPP group. (**Figure 2A**). Wilcoxon test results further confirmed significant differences between the two groups (P < 0.01) at the genus level, with *Faecalibacterium* significantly enriched and *Anaerotruncus* and *Pyramidobacter* significantly decreased in the CPP group (**Figure 2B**). Collectively, these results showed that the increased *Faecalibacterium* and decreased *Anaerotruncus* were important characteristics of the disordered gut microbiota in patients with CPP.

### 3.3 Altered metabolism in CPP patients

**Figure 3:**
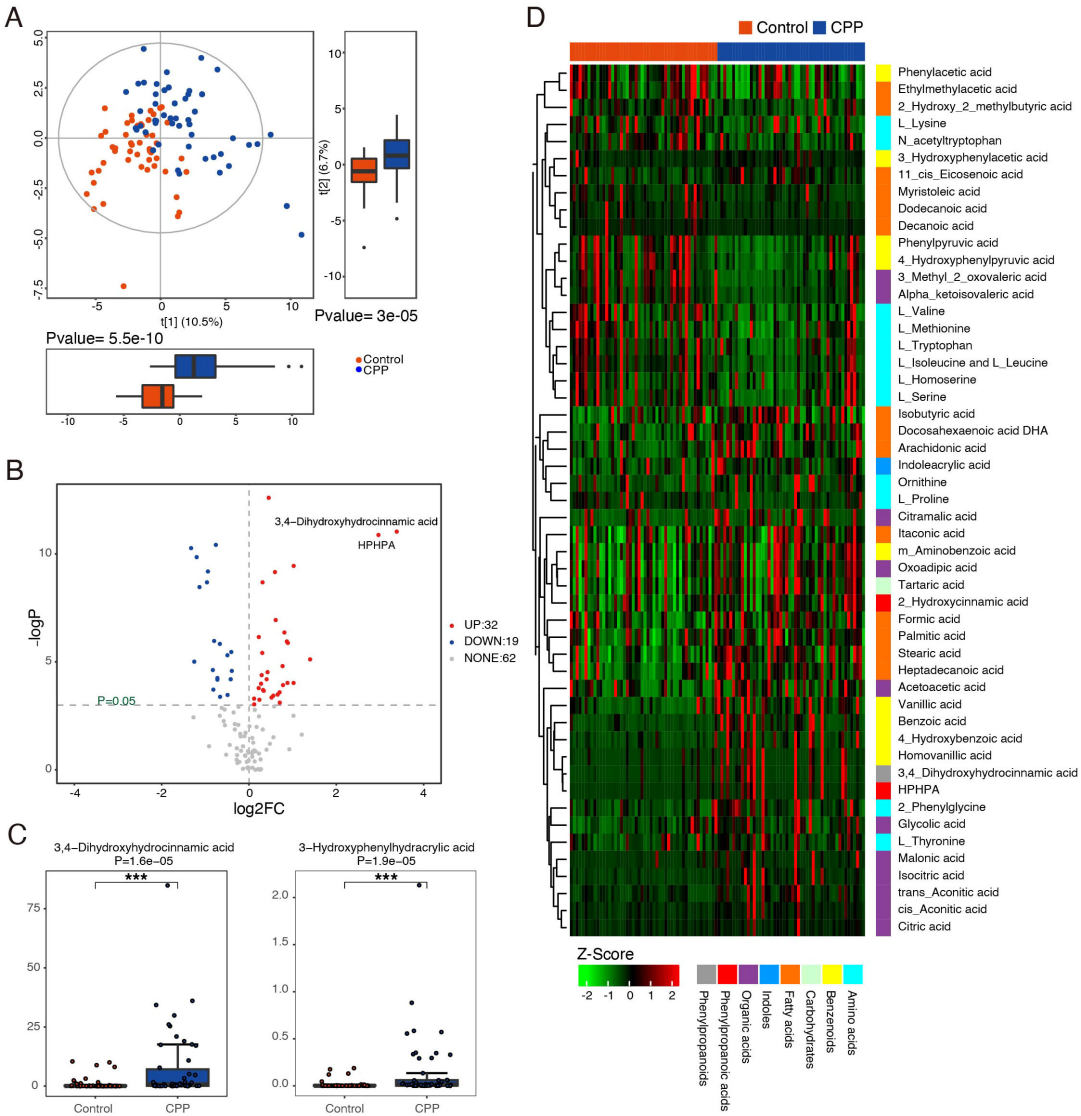
Identification of differential metabolites between the CPP and control groups. **(A)** PLS-DA score plots of the metabolic profiles from the CPP and control groups. Blue represents the CPP group, and red represents the control group. **(B)** Volcano map of differential metabolites. Metabolites with a *P* value < 0.05 and an absolute value of log2FC > 0 were considered significantly different. The red dots (right side) represent metabolites that were increased in the CPP group, and blue dots (left side) indicate those that were decreased. **(C)** The representative differential metabolites with the highest rank (smaller *P* value and larger FC value) between the CPP group and the control group were 3,4-dihydroxyhydrocinnamic acid and HPHPA ***means P < 0.001. **(D)** Z score heatmap of differential metabolites. The X-axis represents individual samples, and the Y-axis represents the metabolites. The red and blue bands at the top represent the control group and the CPP group, respectively. The relative values represented by colors are displayed at the bottom of the figure, with red indicating higher levels of the metabolite and green indicating lower levels.

In this study, UPLC−MS/MS was used to analyze metabolomic data from stool samples in both the CPP and control groups, aiming to identify the differentially expressed metabolites. First, unsupervised PCA was used to evaluate within-group clustering, detect any outliers, and assess group separation. Then, a supervised analysis method (PLS-DA) was used to reduce the influence of individual variation within each group. The results of PLS-DA showed that there were significant differences in the composition of metabolites between the CPP and control groups (**Figure 3A**).

Furthermore, this study used the Mann−Whitney U test (*P* value and fold change [FC] value), a univariate statistical method, to identify metabolites with significantly different expression between the two groups. As shown in **Figure 3B**, there were differences in the expression of small metabolites between the CPP and control groups. Compared with the control group, a total of 51 differentially expressed metabolites were identified, with 32 showing significant upregulation (*P ≤* 0.05, FC>1) and 19 showing significant downregulation (*P ≤* 0.05, FC>1) in the CPP group. These differentially expressed metabolites included amino acids, benzenoids, carbohydrates, fatty acids, indoles, organic acids, phenyl propanoic acids and phenylpropanoids (**Supplementary Table 1**). Among them, the most representative differentially expressed metabolites in the CPP group, ranked by smallest *P* value and largest FC, were as follows: increased 3-3-hydroxyphenyl-3-hydroxypropanoic acid (HPHPA), 3,4-dihydroxyhydrocinnamic acid, homovanillic acid, 3-hydroxyphenylacetic acid, acetoacetic acid, isocitric acid, cis-aconitic acid, citric acid, formic acid, glycolic acid, and decreased 4-hydroxyphenylpyruvic acid, L-tryptophan, phenylpyruvic acid, and phenylacetic acid. Among these metabolites, the largest fold changes were seen with 3,4-dihydroxyhydrocinnamic acid (FC=10.432) and HPHPA (FC=7.803), both of which had small *P* values (**Supplementary Table 1**, **Figure 3C**). A heatmap (**Figure 3D**) visually clusters these metabolites, suggesting potential inter-metabolite interactions.

**Figure 4:**
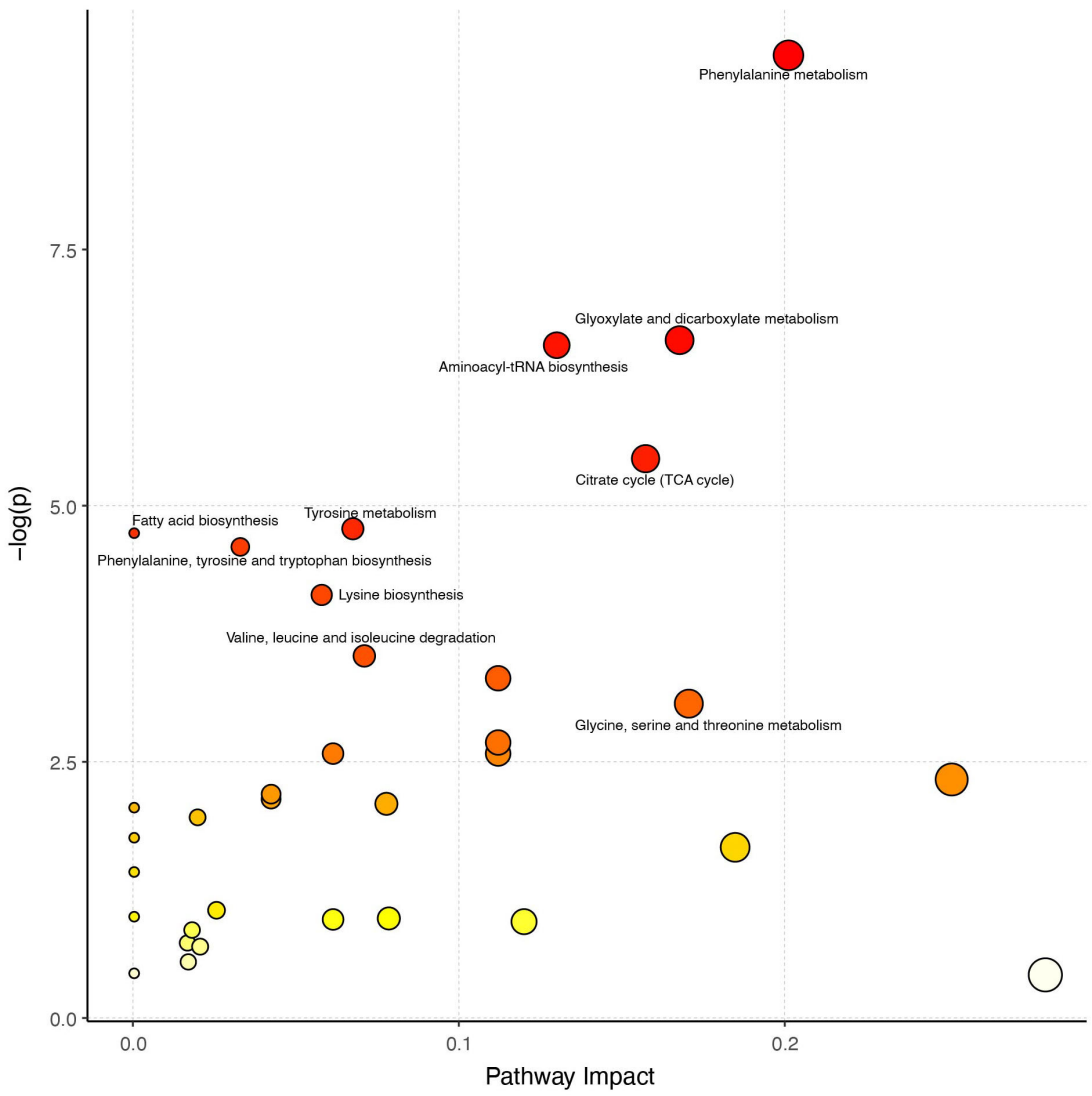
Analysis of metabolic pathways. The larger the circle in the figure, the greater the influence of the metabolic pathway on the grouping. A redder color represents a smaller *P* value, indicating that these pathways warrant greater attention.

Next, we used the hsa library, based on the KEGG database, to perform metabolic pathway enrichment analysis (MPEA) in order to identify the most relevant metabolic pathways associated with these differentially expressed metabolites. Based on the comprehensive *P* value and impact value, the analysis identified several metabolic pathways altered in CPP patients, including phenylalanine metabolism, glyoxylate and dicarboxylate metabolism, aminoacyl-tRNA biosynthesis, citrate cycle (TCA cycle), tyrosine metabolism, phenylalanine, tyrosine and tryptophan biosynthesis, valine, leucine and isoleucine biosynthesis and tryptophan metabolism (**Figure 4**, **Supplementary Table 2**).

### 3.4 Correlation analysis of gut microbiota imbalance and metabolite changes in CPP patients

**Figure 5:**
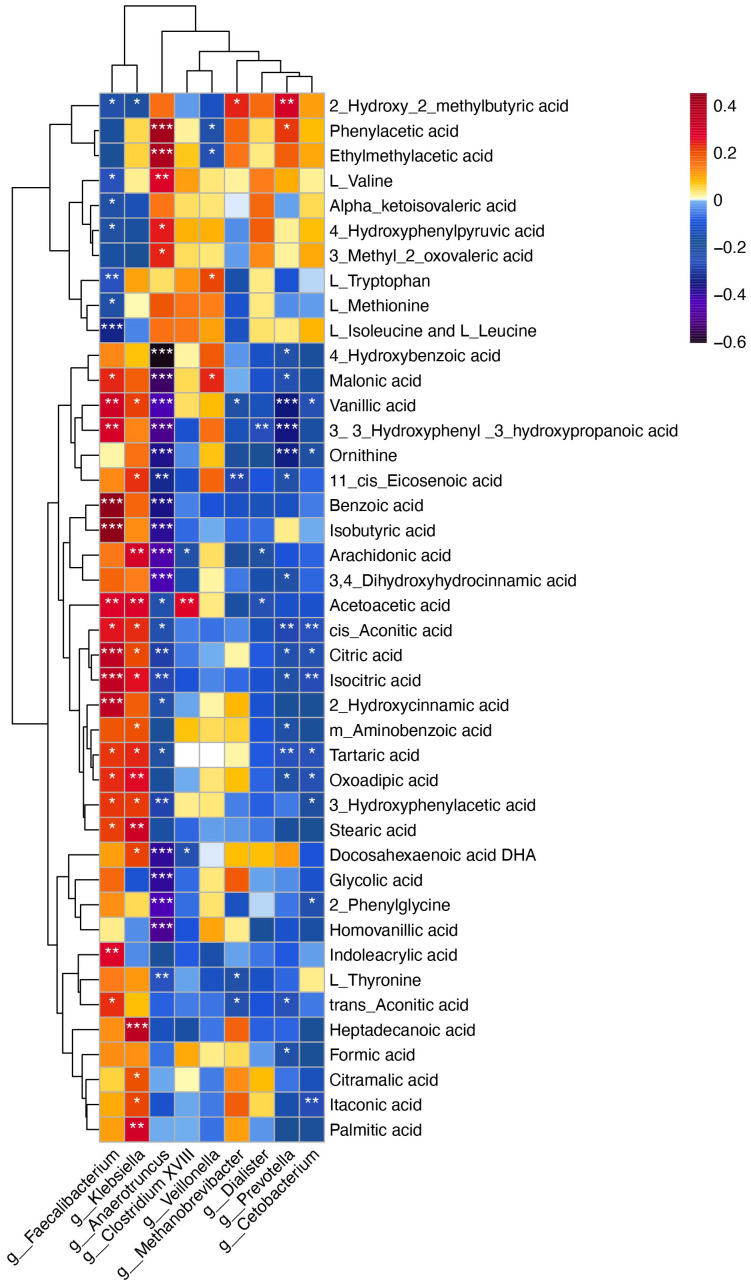
Spearman correlation analysis between differential microbiota at the genus level and differential metabolites. The X-axis represents the microbiota, and the Y-axis represents the metabolites. The color of the grid represents the correlation coefficient of the corresponding metabolite-microbiota. As shown in the figure, warm colors represent positive correlations, while cold colors represent negative correlations. Darker colors indicate stronger correlations. Statistical significance is marked as follows: *P < 0.05, **P < 0.01, ***P < 0.001.

To explore the potential role of the gut microbiome in influencing the onset and progression of CPP through metabolic pathways, we conducted Spearman correlation analysis. The results indicated that several metabolites altered in CPP patients were significantly related to changes in gut microbiota composition. Notably, metabolites involved in phenylalanine and tyrosine biosynthesis and metabolism, including HPHPA, 3,4-dihydroxyhydrocinnamic acid, homovanillic acid, 3-hydroxyphenylacetic acid, and acetoacetic acid, which were significantly increased in CPP patients, showed negative correlations with *Anaerotruncus*. The decreased metabolites 4-hydroxyphenylpyruvic acid and phenylacetic acid exhibited significant positive correlations with *Anaerotruncus* (**Figure 5**).

Additionally, metabolites involved in the TCA cycle and glyoxylate and dicarboxylate metabolism, including isocitric acid, cis-aconitic acid, citric acid, formic acid and glycolic acid, were significantly increased in the feces of CPP patients. Correlation analysis indicated that isocitric acid, cis-aconitic acid and citric acid were positively correlated with *Faecalibacterium*, which were enriched in CPP group, while exhibiting negative correlations with *Anaerotruncus*. Formic acid showed a negative correlation with *Prevotella*, and glycolic acid was negatively correlated with *Anaerotruncus* (**Figure 5**).

Furthermore, L-tryptophan, a crucial metabolite in tryptophan metabolism, was significantly reduced in the feces of CPP patients compared to controls, while oxoadipic acid level was notably elevated. Correlation analysis showed that *Faecalibacterium* was negatively correlated with L-tryptophan and positively correlated with oxoadipic acid (**Figure 5**). Collectively, these above results indicated that the altered metabolites observed in the patients with CPP were correlated with specific gut microbiota profiles.

### 3.5 Correlation analysis of altered gut microbiome and metabolites with serum hormones in CPP patients

We further analyzed the correlation of changed gut microbiota and metabolites with serum hormones, including baseline LH, FSH, E2, and the peak values of LH, FSH, E2 after GnRH stimulation test. The results showed that *Anaerotruncus* was negatively correlated with the peak value of FSH following the GnRH stimulation test, while *Faecalibacterium* exhibited a positive correlation with the peak value of LH after GnRH stimulation test (**Supplementary Figure 1**). Moreover, L-tryptophan showed a negatively correlation with the peak value of LH, and acetoacetic acid showed a positive correlation with basic LH and FSH levels (**Supplementary Figure 2**).

## 4 Discussion

The gut microbiota is a general term for microorganisms residing in the human intestine. The gut microbiota is closely linked to the neuroendocrine system and significantly influences the brain-gut-microbiome axis. Numerous clinical studies have demonstrated bidirectional interactions within the axis. Gut microbes interact with the central nervous system through neural, endocrine, and immune signaling pathways. In turn, the brain can modulate gut microbiota composition and function (16). Recent studies have noted that the gut microbiota in the CPP girls resembles that of obese cohorts (17, 18). Dong et al. identified an association between the gut microbiota in CPP girls and short-chain fatty acids (SCFAs) production (17). Additionally, the gut microbiota and its derived SCFAs have been shown to reverse obesity-induced precocious puberty in female rats by regulating HPG axis (19). Furthermore, the imbalance of the gut microbiota can alter nitric oxide synthesis, which is closely associated with the progression of CPP (20). These studies highlight the gut microbiota as a significant regulatory “organ” of the HPG axis. Different from the previous researches, our study found novel disordered gut microbiota in CPP patients, particularly involving *Faecalibacterium* and *Anaerotruncus*. These changes can influence essential metabolic pathways, including phenylalanine and tyrosine biosynthesis and metabolism, the TCA cycle, glyoxylate and dicarboxylate metabolism, and tryptophan metabolism. Our findings provide valuable insights into the pathogenesis of CPP by integrating specific microbiota profiles and metabolic disruptions.

### 4.1 Distinct gut microbial signature in patients with CPP

Our findings revealed that the most important characteristics of the disordered gut microbiota in patients with CPP were an increased abundance of *Faecalibacterium* and a decreased abundance of *Anaerotruncus*. *Faecalibacterium*, belonging to the phylum *Firmicutes*, resides in the human gut and plays a role in various host metabolic processes. The sole species within this genus, *Faecalibacterium prausnitzii (F. prausnitzii*), functions to produce butyrate. Butyrate, one of the most abundant SCFAs in the colon, serves as an energy source for colonocytes and plays an important role in maintaining intestinal health (21). *F. prausnitzii* has been associated with many endocrine diseases, such as type 2 diabetes and polycystic ovary syndrome, with studies noting significant changes in its abundance in the faces of affected patients (22, 23). While direct studies on the relationship between *F. prausnitzii* and CPP are lacking, researches indicated that its abundance correlate with hormone levels, such as LH and FSH. *F. prausnitzii* could impact the secretion of gut-brain mediators like ghrelin and peptide YY (PYY), by producing SCFAs. Ghrelin is a peptide that can lead to adiposity by enhancing the appetite and reducing fat utilization. PYY, co-localized with GLP-1 in the L-cells of the distal gut. Alterations in ghrelin and PYY levels subsequently impact the secretion of sex hormones (LH, FSH, etc.) by influencing kisspeptin neurons through the HPG axis (23, 24). This finding is consistent with our finding that *Faecalibacterium is* positively correlated with the peak value of LH.

Previous studies have shown that *Anaerotruncus* is associated with Parkinson’s disease, obesity, and other diseases. Zhang et al. found that estrogen deficiency induced by ovariectomy led to an increase in the level of *Anaerotruncus* in the gut of rats (25). These results indicated that there is a certain connection and interaction between serum hormones and *Anaerotruncus*. In our study, we found that the abundance of *Anaerotruncus* in the CPP group was significantly lower than that in the control group, and *Anaerotruncus* was negatively correlated with the peak value of FSH. We speculate that the decreased *Anaerotruncus* might lead to precocious puberty by causing an increase in the level of FSH. However, the mechanism of the *Anaerotruncus*-mediated regulation of HPG axis activation needs to be further explored.

### 4.2 Patients with CPP have different metabolite profiles that are related to different gut microbiota

Through the fecal metabolomic analysis and the correlation analysis with differential gut microbes, this study identified different metabolite profiles and metabolic pathways in CPP patients that were related to specific gut microbiome. First, the biosynthesis and metabolism pathways of phenylalanine and tyrosine were significantly disrupted in the CPP group. This disruption was evidenced by changes in the levels of various metabolites, including HPHPA, 3,4-dihydroxyhydrocinnamic acid, homovanillic acid, 3-hydroxyphenylacetic acid and acetoacetic acid, which were significantly increased, while 4-hydroxyphenylpyruvic acid and phenylacetic acid were decreased in the CPP group. Correlation analysis suggested that these altered metabolites were significantly correlated with *Anaerotruncus*. Our previous study found lower levels of phenylalanine and tyrosine, precursors of catecholamines, alongside higher levels of their major end products (homovanillic acid and vanillylmandelic acid) in the urine samples of CPP subjects compared to healthy controls (15). This suggests that the metabolism of phenylalanine, tyrosine and catecholamines is disordered in children with CPP. Catecholamines are critical neurotransmitters *in vivo* and play an significant role in regulating GnRH secretion by hypothalamic neurons (26–28). In this study, we identified the disordered metabolic pathways of tyrosine and phenylalanine, with a significantly elevated level of homovanillic acid, the major end metabolite of catecholamine, consistent with previous findings. Therefore, we hypothesize that phenylalanine and tyrosine metabolism may regulate GnRH secretion in the hypothalamus by affecting catecholamine metabolism. In addition, among the different metabolites, HPHPA (FC=7.803) and 3,4-dihydroxyhydrocinnamic acid (FC=10.432) exhibited the largest fold changes compared to the control group. HPHPA is an abnormal catabolism product of phenylalanine metabolism in bacteria. Phenylalanine first generates a tyrosine analogue, m-tyrosine, when it is being metabolized by gut microorganisms, and m-tyrosine is further metabolized to generate HPHPA (29, 30). We suggested that HPHPA may act as a catecholamine analogue and potentially modulate catecholamine signaling pathway. However, the precise mechanisms by which HPHPA influences catecholamine signaling pathway remain unclear. Further investigation is required to validate this hypothesis and explore the underlying mechanisms of HPHPA’s role in the pathophysiology of CPP. 3,4-dihydroxyhydrocinnamic acid, also known as dihydrocaffeic acid (DHCA), a metabolic product of gut microorganisms, is known to activate PI3K and Akt phosphorylation, promote insulin secretion, increase the clearance of peripheral glucose, and affect the body’s energy balance (31). There is no direct evidence that 3,4-dihydroxyhydrocinnamic acid is associated with CPP. Considering that energy metabolism is recognized as a significant regulator of the kisspeptin/Kiss1r system (13, 32), we hypothesize that the upregulation of 3,4-dihydroxyhydrocinnamic acid may affect puberty development through its effects on energy metabolism. However, further studies are needed to validate this hypothesis.

Next, we determined that the TCA cycle and glyoxylate and dicarboxylate metabolism pathways were upregulated in CPP patients, as evidenced by altered levels of isocitric acid, cis-aconitic acid, citric acid, formic acid and glycolic acid. Correlation analysis suggested that these changed metabolites were positively correlated with *Faecalibacterium*. The TCA cycle, also known as the citric acid cycle, is essential for energy production. The glyoxylate cycle, which is unique to plants and microorganisms, converts fat into sugar to provide energy and synthesizes dicarboxylic acid to supplement the TCA cycle (33, 34). Energy metabolism plays an important role in the onset of puberty, as it can affect pubertal development through the kisspeptin-Kissr signal pathway. In conditions of excess energy, such as increased food intake, there is an upregulation of Kiss1 mRNA expression in the hypothalamus, leading to elevated LH levels (35, 36). In our previous study, we observed that prepubertal female rats with overnutrition experienced earlier onset of puberty, characterized by decreased expression of ghrelin and increased expression of GnRH and KISS-1/Kisspeptin in the hypothalamus compared to malnourished rats. This suggests a link between energy balance and pubertal development (37, 38). Thus, this study provides further evidence that energy metabolism plays a significant role in the development of CPP.

Additionally, we revealed that L-tryptophan, a key component of the tryptophan metabolism pathway, was significantly lower in CPP patients compared to the control group, and L-tryptophan was negatively correlated with *Faecalibacterium*. Tryptophan is an essential amino acid, which is initially converted to 5-hydroxytryptophan by tryptophan hydroxylase. Subsequently, 5-hydroxytryptophan is further converted to serotonin (5-HT). Tryptophan crosses the blood−brain barrier and plays a crucial role in the synthesis of 5-HT in the central nervous system. 5-HT neurons in the central nervous system can communicate with GnRH neurons through synaptic transmission, where 5-HT binds to various receptors on GnRH neurons, eliciting either inhibitory or excitatory effects. The presence of multiple 5-HT receptors, including 5-HT1A, 5-HT2A, 5-HT2C, 5-HT4, and 5-HT7, allows for complex modulation of GnRH activity in a time- and dose-dependent manner. For instance, binding to the 5-HT1A receptor on the neuronal cell activates Gi protein, resulting in hyperpolarization and inhibition of the rhythmic intracellular GnRH release (39–41). Moreover, previous study found that level of 5-hydroxytryptophan was significantly lower in the urine of CPP subjects, while levels of 5-hydroxyindoleacetic acid and 5-hydroxykynurenamine were elevated, suggesting upregulation of the 5-HT metabolic pathway in the CPP population (15). Our correlation analysis further indicates that L-tryptophan is negatively correlated with peak value of LH. Based on these findings, we hypothesize that gut microbiota in CPP subjects may modulate the tryptophan-5-HT pathway, reducing 5-HT synthesis in the hypothalamus, thus diminishing its inhibitory effect on GnRH neurons. This could lead to increased expression of LH and related sex hormones, facilitating initiation of pubertal sexual development.

## 5 Conclusions

Our study revealed the gut microbial and metabolite characteristics associated with CPP by integrating microbiomics and metabolomics approaches. The most important characteristics of the disordered gut microbiota, at the genus level, were an increased abundance of *Faecalibacterium* and a decreased abundance of *Anaerotruncus*. These gut microbiota changes appear to influence various metabolites and participate in the regulation of several key metabolic pathways, including phenylalanine and tyrosine biosynthesis and metabolism, the TCA cycle, glyoxylate and dicarboxylate metabolism, and tryptophan metabolism. These findings suggest that the gut microbiome may be involved in the onset and progression of CPP through altering the metabolic profile. However, there are some limitations of our study. First, our current findings are purely associative and do not establish a causal relationship between the observed alterations and CPP. Furthermore, we cannot exclude the possibilities that the changes in the gut microbiome and metabolite profiles might be secondary to, or coincident with, CPP or other symptoms associated with the condition. This is hoped to be further investigated. Additionally, this study only included female participants. Given that puberty itself is sexual different, the underlying mechanisms may differ between girls and boys. As such, it is uncertain whether the findings and conclusions of this study are applicable to boys. Future studies are needed to investigate potential sex-specific differences in the mechanisms of CPP. Overall, this study contributes to the understanding of the interaction between gut microbiota metabolomics and CPP, which will be of great significance for the clinical diagnosis and treatment of CPP in the future.

## Data Availability

The datasets presented in this study can be found in online repositories. The names of the repository/repositories and accession number(s) can be found below: https://www.ncbi.nlm.nih.gov/bioproject/924527, PRJNA924527.

## Supplementary material

Supplementary Figure 1 Spearman correlation analysis between changed gut microbiota and serum hormones. LH_base, FSH_base and E2_base refer to the baseline levels of serum sex hormones, while LH_peak, FSH_peak and E2_peak refer to the peak values of serum sex hormones after the GnRH stimulation test. **P* < 0.05, ***P* < 0.01.

Supplementary Figure 2 Spearman correlation analysis between changed metabolites and serum hormones. LH_base, FSH_base and E2_base refer to the baseline levels of serum sex hormones, while LH_peak, FSH_peak and E2_peak refer to the peak values of serum sex hormones after the GnRH stimulation test. **P* < 0.05.
