# Supplementary figures and images for: Metabolomic disorders caused by an imbalance in the gut microbiota are associated with central precocious puberty
Source: Front Endocrinol (Lausanne). 2024 Dec 2;15:1481364. doi: 10.3389/fendo.2024.1481364 (PMC11646730; doi:10.3389/fendo.2024.1481364)

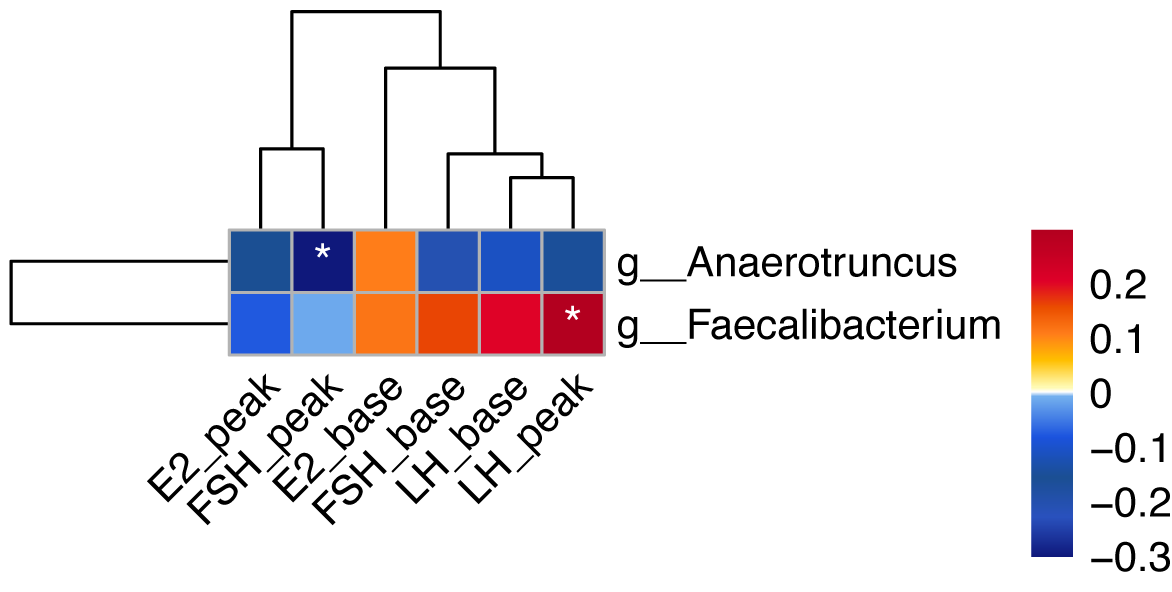

Supplement: Supplementary Figure 1 — Spearman correlation analysis between changed gut microbiota and serum hormones. LH_base, FSH_base and E2_base refer to the baseline levels of serum sex hormones, while LH_peak, FSH_peak and E2_peak refer to the peak values of serum sex hormones after the GnRH stimulation test. *P < 0.05, **P < 0.01. [file Image1.tif]

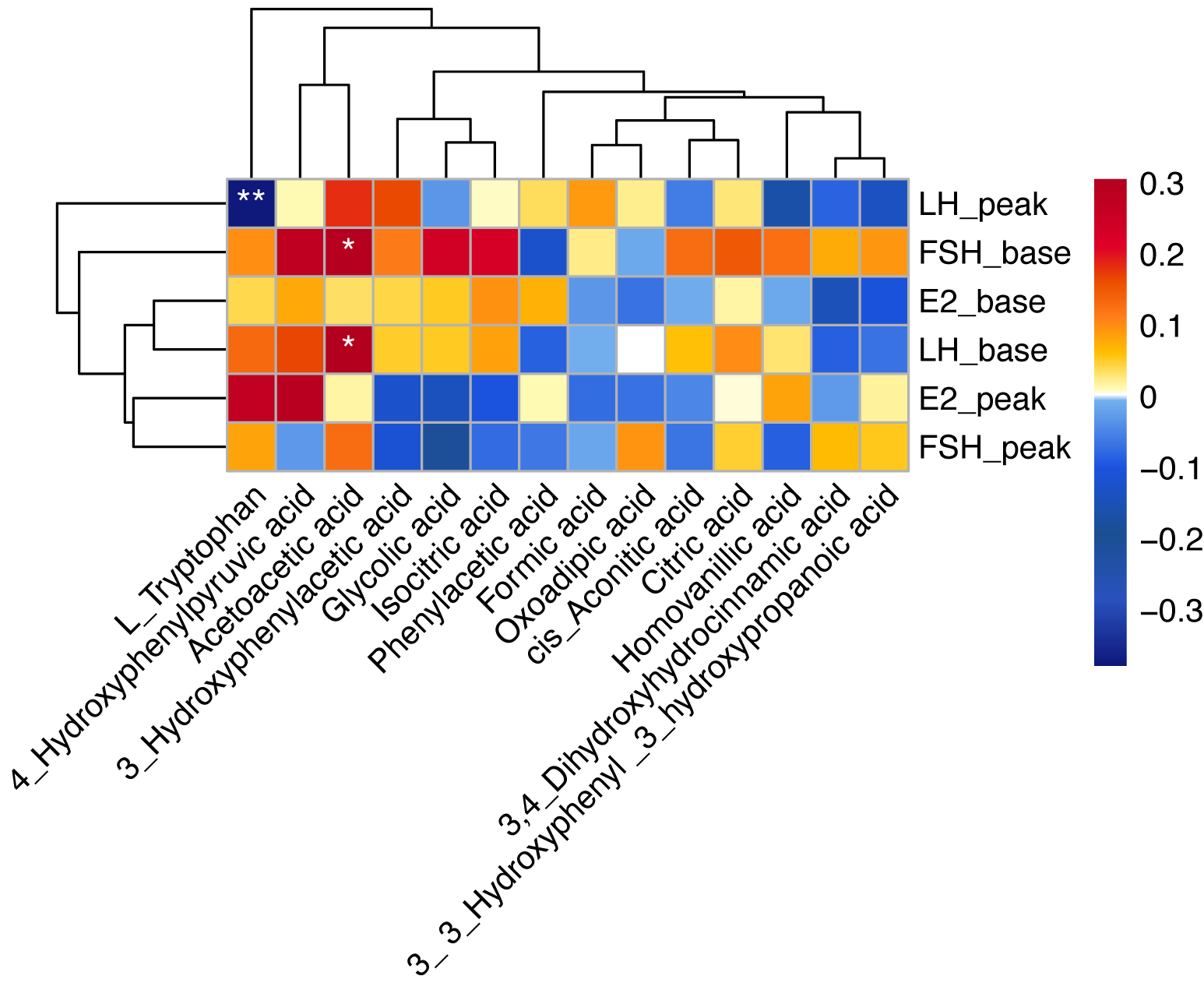

Supplement: Supplementary Figure 2 — Spearman correlation analysis between changed metabolites and serum hormones. LH_base, FSH_base and E2_base refer to the baseline levels of serum sex hormones, while LH_peak, FSH_peak and E2_peak refer to the peak values of serum sex hormones after the GnRH stimulation test. *P < 0.05. [file Image2.tif]
