# Supplementary material for: Metabolomic disorders caused by an imbalance in the gut microbiota are associated with central precocious puberty
Source: Front Endocrinol (Lausanne). 2024 Dec 2;15:1481364. doi: 10.3389/fendo.2024.1481364 (PMC11646730; doi:10.3389/fendo.2024.1481364)
Supplement: Supplementary file 3 [file Table1.docx]

**Supplementary material**

**Supplementary table 1. Differential metabolites between CPP group and Control group.**

| **Class** | **Name** | **HMDBID** | **KeggID** | ***P* value** | **FC** |
| --- | --- | --- | --- | --- | --- |
| Organic acids | Isocitric acid | HMDB00193 | C00311 | 0.0000034 | 1.367 |
| Phenylpropanoids | 3,4_Dihydroxyhydrocinnamic acid | HMDB00423 | C10447 | 0.000016 | 10.432 |
| Phenylpropanoic acids | 3_ 3_Hydroxyphenyl _3_hydroxypropanoic acid | HMDB02643 | NA | 0.000019 | 7.803 |
| Amino acids | L_Isoleucine and L_Leucine | HMDB00172 | C00407 | 0.00003 | 0.591 |
| Organic acids | 3_Methyl_2_oxovaleric acid | HMDB00491 | C03465 | 0.000035 | 0.399 |
| Organic acids | Alpha_ketoisovaleric acid | HMDB00019 | C00141 | 0.000053 | 0.436 |
| Benzenoids | Vanillic acid | HMDB00484 | C06672 | 0.000079 | 2.031 |
| Benzenoids | 4_Hydroxyphenylpyruvic acid | HMDB00707 | C01179 | 0.0001 | 0.521 |
| Fatty acids | Stearic acid | HMDB00827 | C01530 | 0.00011 | 1.51 |
| Organic acids | cis_Aconitic acid | HMDB00072 | C00417 | 0.00017 | 1.24 |
| Benzenoids | Phenylpyruvic acid | HMDB00205 | C00166 | 0.00017 | 0.513 |
| Amino acids | L_Valine | HMDB00883 | C00183 | 0.00021 | 0.456 |
| Fatty acids | Heptadecanoic acid | HMDB02259 | NA | 0.00097 | 1.528 |
| Benzenoids | Benzoic acid | HMDB01870 | C00539 | 0.0017 | 1.754 |
| Carbohydrates | Tartaric acid | HMDB00956 | C00898 | 0.0021 | 1.168 |
| Fatty acids | Decanoic acid | HMDB00511 | C01571 | 0.0026 | 0.575 |
| Organic acids | trans_Aconitic acid | HMDB00958 | C02341 | 0.0026 | 1.823 |
| Fatty acids | 11_cis_Eicosenoic acid | HMDB02231 | C16526 | 0.0028 | 1.853 |
| Amino acids | L_Serine | HMDB00187 | C00065 | 0.0029 | 0.63 |
| Amino acids | L_Methionine | HMDB00696 | C00073 | 0.0043 | 0.755 |
| Organic acids | Citric acid | HMDB00094 | C00158 | 0.0045 | 1.234 |
| Amino acids | L_Tryptophan | HMDB00929 | C00078 | 0.005 | 0.71 |
| Benzenoids | Homovanillic acid | HMDB00118 | C05582 | 0.006 | 2.638 |
| Fatty acids | 2_Hydroxy_2_methylbutyric acid | HMDB01987 | NA | 0.0067 | 0.419 |
| Amino acids | 2_Phenylglycine | HMDB02210 | NA | 0.0082 | 1.703 |
| Fatty acids | Myristoleic acid | HMDB02000 | C08322 | 0.0098 | 0.563 |
| Amino acids | L_Proline | HMDB00162 | C00148 | 0.01 | 0.761 |
| Fatty acids | Formic acid | HMDB00142 | C00058 | 0.011 | 1.342 |
| Benzenoids | 3_Hydroxyphenylacetic acid | HMDB00440 | C05593 | 0.012 | 1.223 |
| Amino acids | L_Lysine | HMDB00182 | C00047 | 0.014 | 0.606 |
| Organic acids | Glycolic acid | HMDB00115 | C03547 | 0.015 | 1.323 |
| Amino acids | N_acetyltryptophan | HMDB13713 | NA | 0.015 | 0.748 |
| Fatty acids | Dodecanoic acid | HMDB00638 | C02679 | 0.015 | 0.605 |
| Fatty acids | Arachidonic acid | HMDB01043 | C00219 | 0.018 | 2.028 |
| Benzenoids | 4_Hydroxybenzoic acid | HMDB00500 | C00156 | 0.018 | 1.841 |
| Organic acids | Malonic acid | HMDB00691 | C04025 | 0.019 | 1.21 |
| Organic acids | Acetoacetic acid | HMDB00060 | C00164 | 0.02 | 1.716 |
| Organic acids | Oxoadipic acid | HMDB00225 | C00322 | 0.023 | 1.166 |
| Fatty acids | Ethylmethylacetic acid | HMDB02176 | C18319 | 0.024 | 0.568 |
| Indoles | Indoleacrylic acid | HMDB00734 | NA | 0.025 | 1.25 |
| Fatty acids | Palmitic acid | HMDB00220 | C00249 | 0.026 | 1.263 |
| Fatty acids | Docosahexaenoic acid | HMDB02183 | C06429 | 0.028 | 1.624 |
| Amino acids | Ornithine | HMDB00214 | C00077 | 0.031 | 1.576 |
| Amino acids | L_Homoserine | HMDB00719 | C00263 | 0.031 | 0.712 |
| Organic acids | Citramalic acid | HMDB00426 | C00815 | 0.032 | 1.47 |
| Benzenoids | Phenylacetic acid | HMDB00209 | C07086 | 0.034 | 0.632 |
| Amino acids | L_Thyronine | HMDB00667 | NA | 0.035 | 1.429 |
| Fatty acids | Itaconic acid | HMDB02092 | #N/A | 0.037 | 1.084 |
| Phenylpropanoic acids | 2_Hydroxycinnamic acid | HMDB02641 | C01772 | 0.039 | 1.18 |
| Fatty acids | Isobutyric acid | HMDB01873 | C02632 | 0.044 | 1.629 |
| Benzenoids | m_Aminobenzoic acid | HMDB01891 | NA | 0.048 | 1.086 |
