# Supplementary material for: Metabolomic disorders caused by an imbalance in the gut microbiota are associated with central precocious puberty
Source: Front Endocrinol (Lausanne). 2024 Dec 2;15:1481364. doi: 10.3389/fendo.2024.1481364 (PMC11646730; doi:10.3389/fendo.2024.1481364)
Supplement: Supplementary file 4 [file Table2.docx]

**Supplementary material**

**Supplementary table 2. Results of metabolic pathway analysis.**

| **Pathway Name** | **Total_In_**  **Pathway** | **Hits** | **Raw *P*** | **Impact** | **Enriched_Compounds** |
| --- | --- | --- | --- | --- | --- |
| Phenylalanine metabolism | 45 | 6 | 8.35E-05 | 0.2 | 3-Hydroxyphenylacetic acid |
|  |  |  |  |  | 4-Hydroxybenzoic acid |
|  |  |  |  |  | Benzoic acid |
|  |  |  |  |  | Phenylacetic acid |
|  |  |  |  |  | Phenylpyruvic acid |
|  |  |  |  |  | trans-2-Hydroxycinnamate |
|  |  |  |  |  |  |
| Glyoxylate and dicarboxylate metabolism | 50 | 5 | 0.0013456 | 0.16666 | cis-Aconitic acid |
|  |  |  |  |  | Citric acid |
|  |  |  |  |  | Formic acid |
|  |  |  |  |  | Glycolic acid |
|  |  |  |  |  | Isocitric acid |
| Aminoacyl-tRNA biosynthesis | 75 | 6 | 0.0014132 | 0.12904 | L-Lysine |
|  |  |  |  |  | L-Methionine |
|  |  |  |  |  | L-Proline |
|  |  |  |  |  | L-Serine |
|  |  |  |  |  | L-Tryptophan |
|  |  |  |  |  | L-Valine |
| Citrate cycle (TCA cycle) | 20 | 3 | 0.0042778 | 0.15625 | cis-Aconitic acid |
|  |  |  |  |  | Citric acid |
|  |  |  |  |  | Isocitric acid |
| Tyrosine metabolism | 76 | 5 | 0.00847 | 0.06666 | 3,4-Dihydroxyhydrocinnamic acid |
|  |  |  |  |  | 3-Hydroxyphenylacetic acid |
|  |  |  |  |  | 4-Hydroxyphenylpyruvic acid |
|  |  |  |  |  | Acetoacetic acid |
|  |  |  |  |  | Homovanillic acid |
| Fatty acid biosynthesis | 49 | 4 | 0.0088272 | 0 | Capric acid |
|  |  |  |  |  | Dodecanoic acid |
|  |  |  |  |  | Palmitic acid |
|  |  |  |  |  | Stearic acid |
| Phenylalanine, tyrosine and tryptophan biosynthesis | 27 | 3 | 0.010105 | 0.03226 | 4-Hydroxyphenylpyruvic acid |
|  |  |  |  |  | L-Tryptophan |
|  |  |  |  |  | Phenylpyruvic acid |
| Lysine biosynthesis | 32 | 3 | 0.016157 | 0.05714 | L-Homoserine |
|  |  |  |  |  | L-Lysine |
|  |  |  |  |  | Oxoadipic acid |
| Valine, leucine and isoleucine degradation | 40 | 3 | 0.029302 | 0.07017 | Acetoacetic acid |
|  |  |  |  |  | Alpha-ketoisovaleric acid |
|  |  |  |  |  | L-Valine |
| Sulfur metabolism | 18 | 2 | 0.036463 | 0.11111 | L-Homoserine |
|  |  |  |  |  | L-Serine |
| Glycine, serine and threonine metabolism | 48 | 3 | 0.046725 | 0.16949 | L-Homoserine |
|  |  |  |  |  | L-Serine |
|  |  |  |  |  | L-Tryptophan |
| Cysteine and methionine metabolism | 56 | 3 | 0.068245 | 0.11112 | L-Homoserine |
|  |  |  |  |  | L-Methionine |
|  |  |  |  |  | L-Serine |
| Pantothenate and CoA biosynthesis | 27 | 2 | 0.076018 | 0.0606 | Alpha-ketoisovaleric acid |
|  |  |  |  |  | L-Valine |
| Valine, leucine and isoleucine biosynthesis | 27 | 2 | 0.076018 | 0.11111 | Alpha-ketoisovaleric acid |
|  |  |  |  |  | L-Valine |
| Synthesis and degradation of ketone bodies | 6 | 1 | 0.098045 | 0.25 | Acetoacetic acid |
| Methane metabolism | 34 | 2 | 0.11287 | 0.04167 | Formic acid |
|  |  |  |  |  | L-Serine |
| Propanoate metabolism | 35 | 2 | 0.11846 | 0.04166 | Acetoacetic acid |
|  |  |  |  |  | L-Valine |
| Ubiquinone and other terpenoid-quinone biosynthesis | 36 | 2 | 0.12412 | 0.07692 | 4-Hydroxybenzoic acid |
|  |  |  |  |  | 4-Hydroxyphenylpyruvic acid |
| D-Arginine and D-ornithine metabolism | 8 | 1 | 0.12859 | 0 | Ornithine |
| Nitrogen metabolism | 39 | 2 | 0.14149 | 0.01923 | Formic acid |
|  |  |  |  |  | L-Tryptophan |
| Biotin metabolism | 11 | 1 | 0.17253 | 0 | L-Lysine |
| Lysine degradation | 47 | 2 | 0.19 | 0.18368 | L-Lysine |
|  |  |  |  |  | Oxoadipic acid |
| Cyanoamino acid metabolism | 16 | 1 | 0.241 | 0 | L-Serine |
| Sphingolipid metabolism | 25 | 1 | 0.35059 | 0.025 | L-Serine |
| Fatty acid elongation in mitochondria | 27 | 1 | 0.37275 | 0 | Palmitic acid |
| Arginine and proline metabolism | 77 | 2 | 0.37992 | 0.07766 | L-Proline |
|  |  |  |  |  | Ornithine |
| beta-Alanine metabolism | 28 | 1 | 0.38356 | 0.06061 | Malonic acid |
| Tryptophan metabolism | 79 | 2 | 0.39223 | 0.11904 | L-Tryptophan |
|  |  |  |  |  | Oxoadipic acid |
| Pyruvate metabolism | 32 | 1 | 0.42499 | 0.01754 | Formic acid |
| Glutathione metabolism | 38 | 1 | 0.4821 | 0.01613 | Ornithine |
| Butanoate metabolism | 40 | 1 | 0.49987 | 0.02 | Acetoacetic acid |
| Fatty acid metabolism | 50 | 1 | 0.58019 | 0.01639 | Palmitic acid |
| Pyrimidine metabolism | 60 | 1 | 0.64788 | 0 | Malonic acid |
| Arachidonic acid metabolism | 62 | 1 | 0.66008 | 0.27869 | Arachidonic acid |

Total_In_Pathway refers to the total number of compounds involved in this pathway; Hits refers to the number of metabolites that actually differ between the two groups in this study; Raw *P* refers to the *P* value calculated by statistical analysis.
